# Pre-Separation Mother–Child Relationship and Adjustment Behaviors of Young Children Left Behind in Rural China: Pathways Through Distant Mothering and Current Mother–Child Relationship Quality

**DOI:** 10.3390/bs14121193

**Published:** 2024-12-13

**Authors:** Ruwen Liang, Karla Van Leeuwen

**Affiliations:** 1 Normal College, Jimei University, Yinjiang Road 183, Xiamen 361021, China; 2 Parenting and Special Education Research Unit, KU Leuven, 3000 Leuven, Belgium; karla.vanleeuwen@kuleuven.be

**Keywords:** left-behind children, mothering, mother–child relationship, rural China

## Abstract

In China, some rural parents do not live together with their children because they migrate to urban regions for work, and therefore they sometimes use a mobile phone in parenting their left-behind children (LBC), who are living with grandparents. This study used a serial mediation model to test the mediating roles of distant mothering and post-separation mother–child relationship quality in the link between recalled pre-separation mother–child relationship quality and social–emotional adjustment of 3-to-6-year-old LBC living in a rural context in China. Cross-sectional questionnaire data were collected from 185 triads, consisting of grandparents (rating child adjustment), migrant mothers (rating mother–child relationship qualities and distant mothering), and preschool teachers (rating child adjustment). The results showed that pre- and post-separation relationship qualities were positively related to each other and to positive distant mothering. There were no serial mediating effects, but a full individual mediating role of post-separation relationship quality and positive distant mothering was identified for the link between child prosocial behavior and externalizing problems, respectively. Despite the general decline in mother–child relationship quality after separation, mothers who perceived a higher quality of the pre-separation mother–child relationship showed a more cohesive relationship with their LBC, which might increase the prosocial behavior of the children. Additionally, a higher quality of the pre-separation relationship was associated with more distant mothering of positive characteristics, which went together with fewer children externalizing problems. These findings highlight the importance of a continuous high-quality mother–child bond and favorable maternal parenting practices in digital interactions for separated families.

## 1. Introduction

Thanks to new information and communication technologies (ICTs), distant parenting practices using mobile phones are now embedded in the lives of families around the world, where children are separated from their parents due to labor mobility, divorce, war, and other situations [1,2,3,4,5,6]. Through instant (e.g., video/audio calls) or asynchronous communications (e.g., social media comments), parents from these families show various forms of support and control, and thus appear to be involved in their children’s development and learning [7,8,9]. Studies showed that, for example, some parents had warm chats with their children to show care or monitored their children’s behaviors [10], while other parents imposed harsh punishments at a distance [11]. These practices are known as “mobile phone parenting” [10] or “remote/distant parenting” [1].

Distant parenting is also a reality in China. In the past three decades, China has witnessed massive rural-to-urban labor mobility and the figure for migrants has soared to 292.51 million in 2021 [12], resulting in the striking phenomenon of “left-behind children” (LBC). Many parents, whose families are registered as rural households, are hindered by financial and institutional regulations (the *hukou* system) from moving with their children to affluent cities [13]. Migrant parents often have no choice but to leave their children behind with their non-migrant parents or grandparents for a long time. A meta-analysis has shown that LBC are more vulnerable to health issues like depression, anxiety, and conduct disorders, compared to children without parental migration [14]. LBC who are separated from their parents at a younger age or who are confronted with the absence of both parents tend to have a higher risk of adjustment behavior problems and less positive adjustment behavior [15,16,17,18,19], which refer to the psychosocial alterations triggered by changes in life [20]. Therefore, these young children and families deserve more academic and societal attention.

Similar to other parents migrating around the world, Chinese migrant parents of young children are resorting to mobile phone parenting [21,22]. Migrant mothers are often the more active participants in these remote mobile phone parenting practices [8,22,23]. In rural China, fathers generally engage much less in parenting [24], which highlights the importance of distant mothering in the context of family separation. Although negative effects of maternal migration on child development outcomes have been found [25,26], this does not mean that LBC are doomed to be disadvantaged in terms of well-being when there is distant mothering. Because of the independence from family structure and arrangements, positive distant motherhood is a critical part of the ecological environment, in which maternal migration takes place, and it is believed to establish a positive relationship between migrant mothers and their LBC, and to contribute to family well-being [5,10,27]. However, more quantitative research is needed to confirm this hypothesis generated from qualitative or preliminary research. Accordingly, this current study concerns the direct and indirect paths between the quality of the pre-separation mother–child relationship and (mal)adaptive behavior of young LBC through distant mothering or/and the quality of the post-separation mother–child relationship.

### 1.1. Distant Mothering and Mother–Child Relationship Quality

For distant mothering in LBC families in China, Liang et al. conceptualized and validated six dimensions of mobile phone parenting including *responsivity*, *autonomy granting*, *psychological control*, *proactive control*, *directive control*, and *harsh punitive control* [8]. Responsivity suggests maternal awareness of their children’s needs, warm and supportive responses to children, and remote involvement [8]. Autonomy granting is imparted when mothers approve, respect, and encourage their children to be independent and individuated [8]. Psychological control indicates the extent to which mothers interfere with their children’s thoughts, self-expression, and emotional experiences to control them [8]. Proactive control refers to mothers’ democratic, appropriate, and essential management regarding the socialization of their children [8]. Directive control means maternal behavioral control by commanding or demanding children with explicit pressure [8]. Harsh punitive control means maternal dominance over her children through untouched physical punishment or corporal punishment carried out by a co-present caregiver in their telecommunications [8].

Pianta describes parent–child relationships as a certain system involving feelings of closeness, information sharing, memories of the other’s behavioral patterns, and expectations for the other’s reactions [28]. Based on attachment theory, mother–child relationships were conceptualized and operationalized as the positive and negative affective qualities of mother–child interactions such as the levels of closeness or conflict [29]. Mother–child dyads with higher levels of closeness and lower levels of conflict suggest more cohesive mother–child relations, i.e., good quality of mother–child relationships. Undoubtedly, the definition itself shows that mother–child relationship quality is closely related to maternal parenting practices.

In theoretical parenting models, parenting and parent/child characteristics are in reciprocal interaction [30,31], indicating the bidirectional associations between mothering and mother–child relationship quality. Research shows evidence for these theoretically assumed relationships. In a six-year longitudinal study of American adolescents and their divorced mothers, inappropriate maternal discipline and the mother–child relationship quality were not only negatively associated concurrently, but also longitudinally [32]. In Chinese families with young children, Chen et al. found that mothers with authoritative childrearing attitudes and practices reported better mother–child relationships while mothers with authoritarian styles indicated worse relations [33]. These associations have additionally been documented in other research that focused on general parenting [34,35,36].

Besides physically present parenting, Abel et al. argue that remote interactions between family members through ICT can also shape family relationships in a harmonious or conflictual way [7]. Canary and Stafford identified dimensions of long-distance family relationship maintenance practices, namely openness, positivity, assurances, network, and sharing tasks [37]. These five dimensions were positively associated with child-perceived relationship quality in a sample of Chinese migrant parents and their school-aged LBC, with the strongest association for mother–daughter dyads [38]. Nurturing parent–child intimacy can be understood as a purpose and motivation of mobile phone parenting [27]. Based on narratives from migrant mothers, many qualitative researchers agree on the favorable role of distant maternal support in the mother–child bond [1,2,10,39]. In addition to the mother’s perspective, left-behind adolescents in the study by Tang et al. indicated that high-quality telecommunications provided a sense of togetherness and sharing and protected their relationships during prolonged separation [5].

However, negative distant mothering may increase the risk of problematic mother–child relationships. Some researchers have discussed the complexities of mothers’ distant controlling practices [5,6,10,11,39]. They found that migrant mothers tended to view these practices as supportive and a way of protective involvement in their children’s life, while their LBC perceived these maternal behaviors as a form of (excessive) supervision. This misunderstanding and ambivalence made children reluctant to respond to their mothers. Few studies in migrant populations have shown statistically significant associations between distant maternal control and family relationships, while consistent evidence has been found in research on mobile phone parenting among Western non-migrants. For example, children from non-migrant families who perceived more parental monitoring through mobile phones reported poorer family relations [9]. Jensen et al. indicated that the quality of parent–child relationships can fluctuate due to the dynamics between digital support and pressure experienced by children [40]. Nevertheless, these studies primarily focused on older children and their parents. No prior study has investigated whether distant maternal control, such as monitoring, works similarly for the relationships between mothers and younger children. For other intrusively distant mothering, such as psychological control and harsh punitive control, it remains unclear whether and how they relate to mother–child relationship quality.

Given that mother–child relations are not static over time, many believe that relationship quality between mothers and LBC will decrease due to the absence of the migrating mother [41,42], but there is little evidence to support this supposed change. The difference in quality of mother–child relationships before and after the separation still needs to be examined. In addition, because of the life changes in migrant families and mothers’ different ways of parenting, mother–child relationship could change. An additional question is therefore to what extent distant parenting plays a role in the association between pre-separation and post-separation mother–child relationships. This is relevant for different reasons. First, strong mother–child relationships before separation are fundamental for later closeness. Attachment theories have indicated that child early attachment security is a predictor of subsequent interactions with their mother [43,44]. Also, Madianou and Miller (2011) found that migrant mothers and LBC struggled to grow closer if early ties were poorly forged [10]. Second, weak mother–child closeness may be related to later maternal neglectful parenting or other negative mothering behaviors while close relationships, tend to be linked to subsequent positive mothering [45,46,47]. As Chen pointed out, mobile phone parenting is intertwined with the fluctuation of family relationships at different stages [48]. The mother–child relationship is one of the elements that migrant mothers often use to construct their motherhood and responsibilities [22]. Third, jumping back to the aforementioned evidence, maternal parenting practices can predict the subsequent mother–child relationship quality [32]. Therefore, integrating the three pathways among earlier and later qualities of the mother–child relationship as well as maternal parenting, it is reasonable to speculate that the quality of pre-separation mother–child relationships is possibly associated with the quality of post-separation relationships through distant mothering as a mediator.

### 1.2. Mother–Child Relationship Quality and Child Adjustment

Mother–child relationship quality at different times also matters for a child’s psychosocial development. In the context of parental migration, LBC need to psychosocially adapt to changed living conditions after their parents have left. The child behavior resulting from these adjustments [20] is often operationalized in research as externalizing/internalizing problems and socioemotional competence. Externalizing problems in early childhood generally appear as aggressive behaviors and attention deficits while early internalizing problems usually take the form of emotional difficulties, such as negative affect, anxiety, and withdrawal [49].

Studies on LBC have devoted much attention to the concurrent associations between the quality of mother–child relations and child adjustment. Children with a more cohesive mother–child relationship usually show both fewer internalizing difficulties like loneliness and depression [50,51,52] and fewer externalizing difficulties like hyperactivity symptoms [53,54]. However, the issue should not be viewed solely from a deficiency point of view. A more comprehensive perspective has led to more research into how mother–child relationships play a role in LBC well-being and skills. In particular, intimate bonds between migrant mothers and LBC are positively associated with children’s psychological resilience [55], satisfaction of psychological needs [50], prosocial behaviors [54], and social competence (e.g., interpersonal skills, self-management skills, and academic skills) [56].

Apart from the cross-sectional associations, earlier mother–child relationships are also related to later child adjustment behaviors in migrant families. For instance, in their longitudinal study among LBC aged 8 to 11 years, Wu et al. found that mother–child closeness negatively predicted child internalizing and externalizing problems after six months, but it was not a significant predictor for child prosocial skills [54]. Another study found that shy left-behind adolescents with strong parent–child cohesion, measured one year before, reported fewer depressive symptoms [57]. These studies, however, only investigated the quality of the parent–child relationships after the LBC had been separated from their parents. They fail to demonstrate the pre- and post-separation changes in mother–child closeness and how the quality of the mother–child relationships at different times is related to the child’s adjustment. Considering the reciprocal paths between mother–child relations and remote mothering, it seems fair to assume both direct and indirect links between pre-separation mother–child relations and child adjustment.

### 1.3. Indirect Associations Among the Qualities of Pre- and Post-Separation Mother–Child Relationships, Distant Mothering, and Child Adjustment

Mothering may play a mediating role between earlier mother–child relationship quality and later child adaptive behaviors. Not much research has explored this pathway, but one study provided evidence that maternal negative control partially mediated the effect of the quality of the mother–child bond on externalizing difficulties of young adolescents [58]. The potential mechanisms can be disassembled into two parts. First, as we reviewed earlier, earlier mother–child relationship quality can be a significant predictor for later mothering practices [32,59,60]. Second, meta-analyses have confirmed that increased child internalizing and externalizing symptoms are associated, although weakly, with lower levels of parental warmth, behavioral control, and autonomy granting and with higher levels of harsh control and psychological control [61,62]. With regard to children’s competence, another meta-analysis found that children show more prosocial behavior when parenting is warmer, more reasonable, and positive (*r* = 0.17) on the one hand, and less hostile, harsh, and negative (*r* = −0.11) on the other [63]. Although a few researchers identified associations between the intensity/frequency of parent–child remote communications and LBC’s well-being [64,65], little is known about how mothering through mobile phones is associated with various adjustment behaviors of LBC. Combining the two pathways, it is justifiable to propose a simple mediation model where distant mothering mediates the association between the quality of the pre-separation mother–child relationship and child adjustment.

Also, current mother–child relationship quality can account for the associations between maternal parenting practices and child adjustment. According to attachment theories, children’s early interactive experiences with the primary caregiver can be represented as internal working models of the caregiver–child relationship [66]. The internalized representation of the caregiver–child relationship then influences children’s emotion regulation process, children’s evaluation and expectancy of themselves and others, their representation of social relationships, and motivational functioning, which may further cause or protect children from psychopathology in childhood [67]. Basically, DeKlyen and Greenberg concluded that parenting and attachment (including post-infancy mother–child relationships) can be protective or risk factors for child psychopathology, and the associations can also be mediated by each other [67]. Some studies have applied process models where parent–child relations play a mediating role between certain parenting practices and child adjustment [36,58,68]. For example, Bosmans et al. found that in Belgian Flemish younger adolescents, secure mother–child attachment mediated the associations between maternal negative control and adolescent externalizing difficulties [58]. A similar pathway was also examined in Chinese preschoolers and their parents by Zhang et al. with parent–child relations as a mediator linking mindful parenting and child emotional adaptation [36].

Further, given DeKlyen and Greenberg’s interpretations of parenting and attachment as mutual mediators [67], the mediator chain can be extended if we consider both pre- and post-separation mother–child relations. That is to say, distant mothering and post-separation relation quality are surmised to successively mediate the associations between the quality of pre-separation mother–child relations and child adjustment.

### 1.4. The Current Study

Taking together the above arguments and evidence, the current study primarily used a serial mediation approach to examine the associations among pre-separation mother–child relationships, distant mothering, current mother–child relationships, and child adjustment among young LBC and their families in China. Financial and human resources did not allow us to adopt a longitudinal design; therefore, all data were collected at the same point in time. Regarding pre-separation mother–child relationships, we used a retrospective approach that has been used in previous studies [69,70], by asking mothers about their memories of the quality of their relationship with their child before the separation.

Based on prior research [41,42], we proposed Hypothesis 1: The quality of the migrant mother–LBC relationship would be significantly higher before the separation compared to after the separation.

**Figure 1:**
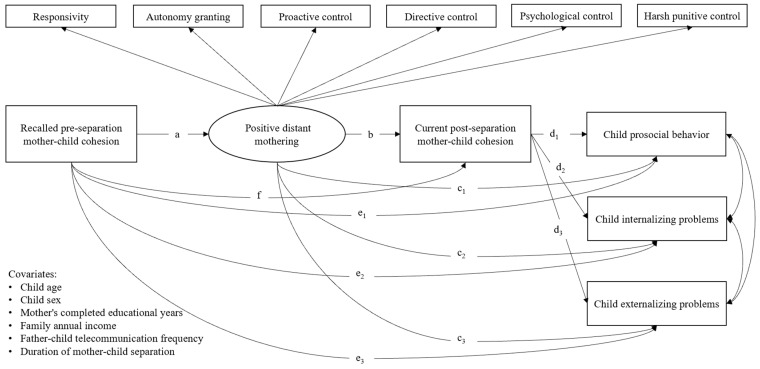
Hypothetical serial mediation model regarding the associations between recalled pre-separation mother–child cohesion, positive distant mothering, current post-separation mother–child cohesion, and child adjustment.

Next, we evaluated several hypotheses using a serial mediation model (see Figure 1).

With respect to the direct associations, based on previous studies [52,54,56], we formulated Hypothesis 2: Higher quality of both pre-separation and current mother–child relationships (i.e., mother–child cohesions) would be related with fewer child internalizing and externalizing difficulties and more prosocial behavior, respectively.

Additionally, in light of previous findings [61,62], positive distant maternal parenting was hypothesized to independently explain variations in child outcomes as Hypothesis 3: Fewer child difficulties and more prosocial behaviors would be present if the migrant mothers reported higher levels of positive mothering, operationalized by more responsivity, autonomy granting, proactive control, directive control, as well as less psychological and harsh punitive control.

Considering the results of previous studies [36,58,67,68], we put forward Hypothesis 4: The associations between the quality of pre-separation relations and child adjustment would be serially or independently mediated by positive distant mothering and the quality of current mother–child relationships (see Figure 1). As stated in Hypothesis 4, we expected that a better quality of pre-separation relations would be associated with higher levels of positive mothering, which in turn would be associated with current mother–child cohesion, serially linked to fewer child internalizing and externalizing difficulties and more prosocial behavior.

## 2. Methods

### 2.1. Participants and Procedures

In total, 185 young left-behind children, along with their families and teachers, were included in the present study. The research was conducted consistent with recognized ethical guidelines by the Social and Societal Ethics Committee of KU Leuven (Approval number: G-2021-4012-R2(MAR)). With the approval and assistance of local preschool leaders or teachers, target families were invited and recruited from 32 rural preschools with similar childcare quality in Chuxiong Yi Autonomous Prefecture, Yunnan, a less-developed province in southwest China with considerable labor migration. Inclusion criteria were families (a) having at least one child (3 to 6 years old) left behind by both migrant parents for more than three months; (b) with in-hometown grandparents as a primary caregiver; (c) regularly using mobile phone for remote mother–child interaction; and (d) with grandparents and mothers who are able to respond to questionnaires or interviews. All questionnaires were administered remotely due to COVID-19 restrictions. Data were collected between May 2022 and January 2023. The data collection for a specific child and their family was ensured to finish within three months. We initially received 216 questionnaires reported by teachers. Twenty-two triads among these participants did not complete either mother questionnaires or grandparent questionnaires. Eighteen teacher questionnaires involved paired siblings from the same families, but only one of the siblings was randomly selected and grandparents and mothers completed questionnaires regarding one child. We thus excluded the triad data with these two types of missingness. All participants received remuneration for their contribution. Table 1 shows the characteristics of the included 185 children and their families. In addition to the features listed in Table 1, some family information is provided here for supplementary context. Around 80.55% of the grandparent questionnaires were reported by grandmothers. Among the migrant mothers (*M*_age_ = 31.94, *SD* = 3.76; (re)married mothers: 71.35%; divorced mothers: 23.75%; widowed mothers: 4.86%), 91.35% had occupations like non-technical, semi-technical, or technical workers, and 89.73% communicated with their children remotely at least three times a week. Most children (67.57%) experienced their first long-term separation from their migrant mothers after 3 years old.

### 2.2. Measures

#### 2.2.1. Child Adjustment

Child adjustment was measured with the Chinese version of the Strength and Difficulties Questionnaire (SDQ), a brief behavioral screening questionnaire for 3–16-year-olds [71]. Both teacher- and caregiver-reported SDQ have been validated by Du et al. among Chinese families [72]. The 25-item questionnaire is composed of five subscales: (a) emotional symptoms (five items, e.g., “Many worries, often seems worried”); (b) conduct problems (five items, e.g., “Often lies or cheats”); (c) hyperactivity/inattention (five items, e.g., “Constantly fidgeting or squirming”); (d) peer relationship problems (five items, e.g., “Gets on better with adults than with other children”); and (f) prosocial behavior (five items, e.g., “Kind to younger children”). We alternatively adopted a three-subscale division including (a) internalizing problems (10 items including emotional symptoms and peer relationship problems); (b) externalizing problems (10 items including conduct problems and hyperactivity/inattention); and (c) prosocial behavior (five items). Considering the poor Cronbach’s alphas in previous studies, Bøe et al. recommend adopting ordinal alpha to decide whether the internal consistency of the subscales of SDQ is acceptable [73]. The ordinal Cronbach’s alphas, calculated with the R-package “misty” [74] were 0.75 and 0.66 for internalizing problems, 0.62 and 0.79 for externalizing problems, and 0.70 and 0.80 for prosocial behavior, for teacher- versus grandparent-reported measures, respectively. Respondents answered all items on a three-point rating scale ranging from 0 (*not true*) to 1 (*somewhat true*) to 2 (*certainly true*). This questionnaire was completed digitally by preschool teachers for each child whose family was included in this study, and answered by an in-hometown primary caregiver (grandparent) from each family through telephone interviews conducted by investigators. The total scores for internalizing problems, externalizing problems, and prosocial behavior were calculated separately by averaging the sum of teachers’ ratings and grandparents’ ratings, with higher scores suggesting more difficulties or behavior. The correlations between teacher-reported and grandparent-reported child outcomes were 0.35 (*p* < 0.001), 0.50 (*p* < 0.001), and 0.25 (*p* < 0.001), respectively, for prosocial behavior, internalizing problems, and externalizing problems.

#### 2.2.2. Distant Mothering

Distant mothering was assessed by the Mobile Phone Parenting Practices Questionnaire (MPPPQ), which was developed and validated among rural–urban migrant parents with young children in China [75], using a five-point response format (1 = *never* to 5 = *always*). The 47-item questionnaire involves six mobile phone parenting dimensions: (a) responsivity (19 items, e.g., “I watch my child doing his/her stuff in silence but as a companion”, *α* = 0.95); (b) autonomy granting (five items, e.g., “I encourage my child to tackle problems by him/herself”, *α* = 0.89); (c) psychological control (six items, e.g., “I compare my child with his/her better-performed peers”, *α* = 0.85); (d) proactive control (eight items, e.g., “I ask my child whether he/she has regular meals or a balanced diet”, *α* = 0.93); (e) directive control (five items, e.g., “I ask my child to be obedient to the in-hometown caregiver”, α = 0.81); and (f) harsh punitive control (four items, e.g., “I remotely ask my child to punitively stand”, *α* = 0.85). The mean score of each subscale was calculated, with a higher mean score indicating more frequent parenting practices that are part of the parenting dimension. Migrant mothers received and completed the electronic versions of the MPPPQ via short messaging.

#### 2.2.3. Quality of Recalled Pre-Separation and Current Post-Separation Mother–Child Relationships

The two variables were both assessed by adopting the Child–Parent Relationship Scale—Short Form (15 items), applicable for children aged 3 to 12 [29]. The Chinese version of this scale was translated, adapted, and validated by Deng [76]. The scale consists of two domains: (a) conflicts (seven items, e.g., “My child’s feelings toward me can be unpredictable or can change suddenly”) and (b) closeness (eight items, e.g., “If upset, my child will seek comfort from me”). The items are reported on five-point Likert scales ranging from 1 (*definitely does not apply*) to 5 (*definitely applies*). Mothers in this study were asked to report the recalled pre-separation and current post-separation mother–child relationships separately. Mother–child cohesion was operationalized by first reversing the conflicts subscale score and then summing all of the ratings, with higher total scores indicating better quality of recalled pre-separation or current post-separation mother–child relationships. The Cronbach’s alphas were 0.83 and 0.85 for recalled pre-separation and current post-separation cohesion, respectively. The questionnaire was completed digitally by migrant mothers.

#### 2.2.4. Covariates

A previous study found that the average paternal involvement in rural China was quite low but could still influence early childhood development [77]. We thus controlled for distant fathering, which was indexed by the frequencies of contact with fathers per week. Moreover, maternal education levels [26], family income [17], and child age and sex [61,62] are important variables when investigating parenting and child adjustment. In separated families, the duration of mother–child separation also accounts for child behavioral outcomes [17,19]. Demographic variables including child age and sex (0 = *girl*, 1 = *boy*), mother’s completed educational years, family annual income, father–child telecommunication frequency, and duration of mother–child separation were thus all controlled for in the models. These variables were provided in the electronic demographic questionnaire completed by migrant mothers.

### 2.3. Data Analysis

After descriptive and correlation analyses were conducted, we performed a paired samples *t*-test to compare the means of recalled pre-separation and current post-separation mother–child cohesion. To address the principal research question of this study, we used structural equation modeling (SEM) to examine the indirect associations between recalled pre-separation mother–child cohesion and child adjustment through the serial mediators of positive distant mothering and current post-separation mother–child cohesion. First, a measurement model was processed with confirmatory factor analysis (CFA) to test the fit of the second-order latent factor “positive distant mothering”. This latent factor was estimated to consist of six first-order factors including maternal responsivity, proactive control, directive control, autonomy granting, psychological control, and harsh punitive control (see Figure 1). Second, after the measurement model was confirmed, as in Figure 1, we established the structural model, a serial mediation model, to examine the hypothesized pathways among the pre-separation mother–child cohesion (independent variable), positive distant mothering (the first-order mediator), post-separation mother–child cohesions (the second-order mediator), and child internalizing problems/externalizing problems/prosocial behavior (dependent variables) after controlling for the covariates. The indirect paths and their notations are as follows: path 1: abd_1_, path 2: ac_1_, path 3: fd_1_, path 4: abd_2_, path 5: ac_2_, path 6: fd_2_, path 7: abd_3_, path 8: ac_3_, and path 9: ac_3_. The indirect paths were rendered significant when 95% confidence intervals (CIs) did not contain zero.

The SEM procedures were performed with the package “lavaan” in R with maximum likelihood estimation [78]. We evaluated the indexes of fit for the measurement model based on the *χ*^2^/*df,* the root mean square error of approximation (RMSEA), and the standardized root mean square residual (SRMR). The structural model was estimated according to *χ*^2^/*df*, the comparative fit index (CFI), the Tucker-Lewis index (TLI), the RMSEA, and the SRMR. As West et al. suggested fit indices [79], this study used the following five criteria to evaluate the models: (a) *χ*^2^*/df* < 5, (b) CFI > 0.95, (c) TLI > 0.95, (d) RMSEA < 0.06, and (e) SRMR < 0.08.

## 3. Results

### 3.1. Means, Standard Deviations, Correlations, and Paired T-Test

Table 1 presents the descriptive statistics of the key variables. The correlations among them are displayed in Table 2. Child prosocial behavior was positively correlated with pre- and post-separation mother–child cohesion, maternal responsivity, and autonomy granting with small to medium effect sizes according to Cohen’s criteria [80]. Child internalizing problems were negatively correlated with pre- and post-separation mother–child cohesion, responsivity, proactive control, and autonomy granting, and positively correlated with psychological and harsh punitive control with small to medium strength. The correlations regarding child externalizing problems and the effect sizes of the correlations were similar to the bivariate relationships involving internalizing problems, except for the non-significant association with maternal psychological control. Among the demographic variables, children from families with higher annual income and more frequent father–child distant communications showed more prosocial behavior. More internalizing problems were reported for girls, children of less-educated mothers, and children from lower-income families with fewer father–child telecommunications. Besides the correlations regarding child social–emotional outcomes, pre- and post-separation mother–child cohesions were both positively correlated with maternal responsivity, proactive control, and autonomy granting, while psychological and harsh punitive control was negatively correlated with post-separation mother–child cohesion only.

A paired *t*-test was used to examine the change in the quality of the mother–child relationships before and after their separation. The mean of the post-separation mother–child cohesion was significantly smaller than the mean of the recalled pre-separation mother–child cohesion, *t*(184) = 6.64, *p* < 0.001, suggesting a drop in mother–child relationship quality after maternal migration.

### 3.2. SEM Analysis Regarding Mother–Child Cohesion, Distant Mothering, and Child Adjustment

**Figure 2:**
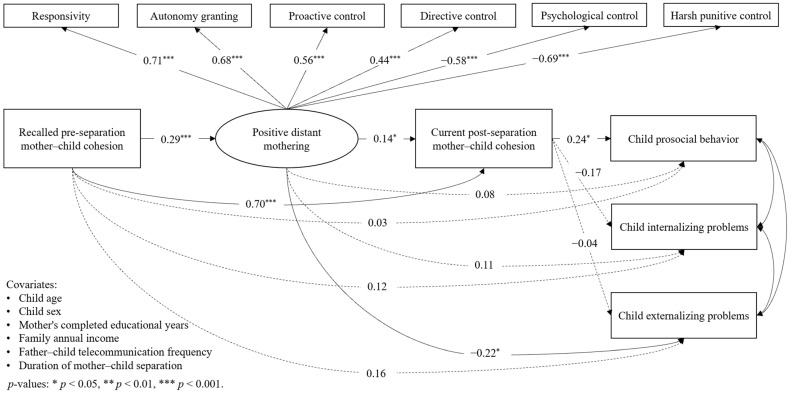
Standardized coefficients for the structural model examining the mediation effect of distant positive mothering and post-separation mother–child cohesion on the associations between pre-separation mother–child cohesion and child outcomes.

In the initial step, the measurement model involving six distant mothering dimensions from the MPPPQ was tested as the latent variable “positive distant mothering”. The measurement model demonstrated a good model fit (*χ*^2^/*df* = 1.04, RMSEA = 0.02, and SRMR = 0.03). As illustrated in Figure 2, the standardized factor loadings for distant mothering dimensions were all acceptable, with the absolute values ranging from 0.44 to 0.71.

In the second step of the SEM analysis, the hypothesized structural model was examined when considering all the demographic covariates that were regressed on all the mediators (i.e., positive distant mothering and post-separation mother–child cohesion) and dependent variables (i.e., prosocial behavior, and internalizing and externalizing problems). This model demonstrated an acceptable model fit (*χ*^2^/*df* = 1.04, CFI = 0.995, TLI = 0.991, RMSEA = 0.02, and SRMR = 0.03). Figure 2 and Table 3 show the overall path coefficients of the direct and mediated associations among the key variables. No serial mediation effects of distant mothering and post-separation cohesion were found for the associations between pre-separation cohesion and child outcomes. Nevertheless, some simple mediation effects were present. Child prosocial behavior was positively associated with post-separation mother–child cohesion (*β_d_*_1_ = 0.24, *p* = 0.031). The direct association between pre-separation cohesion and prosocial behavior was not significant, while post-separation cohesion was a significant mediator between them (*β_fd_*_1_ = 0.16, *p* = 0.033), suggesting that post-separation cohesion statistically played a full mediating role. Child externalizing problems had a direct negative association with positive distant mothering (*β_c_*_3_ = −0.22, *p* = 0.021). Additionally, positive distant mothering fully mediated the association between pre-separation cohesion and externalizing problems (*β_ac_*_3_ = −0.06, *p* = 0.046, *β_total_*_8_ = −0.22, *p* = 0.038). Although the serial mediation effect (i.e., indirect path 7) was insignificant, the total effect combining pre- and post-separation mother–child cohesions and positive distant mothering was significant (*β_total_*_7_ = −0.38, *p* = 0.015). No (in)direct effects were identified for the associations with child internalizing problems, but total effect 6 (i.e., the total effect of the indirect path “pre-separation cohesion—post-separation cohesion—internalizing problems”) was significant (*β_total_*_6_ = −0.24, *p* = 0.001). Besides the links regarding child outcomes, the independent variable and the two hypothesized mediators were significantly associated with each other. Especially, mothers who recalled more cohesive pre-separation mother–child relations reported more positive distant mothering practices (*β_a_* = 0.29, *p* < 0.000) and more cohesive post-separation relations (*β_f_* = 0.70, *p* < 0.000). More positive distant mothering was positively related to more cohesive post-separation relations (*β_b_* = 0.14, *p* = 0.031).

## 4. Discussion

Our research examined the role of maternal mobile phone parenting and mother–child relationship quality in the social–emotional adaption of young LBC in China, a perspective that is seldomly taken when it comes to quantitative inquiries of their family environments. This study found that the quality of mother–child relationships and positive distant mothering practices had a positive association. Although migrant mothers on average perceived a decline in mother–child relationship quality after being separated from their LBC, the post-separation relationship quality was strongly related to the pre-separation relationship quality. An interesting finding is that neither positive distant mothering nor current post-separation mother–child cohesion provided a *direct* explanation for child adjustment, but served as a *pathway* through which pre-separation mother–child cohesion is associated with child prosocial behavior (via post-separation cohesion) and with externalizing problems (via positive distant mothering).

### 4.1. Associations Between Qualities of Mother–Child Relationships and Distant Mothering

To better understand the mediation effects, we first discuss the direct associations among the independent variable (i.e., pre-separation cohesion) and mediators (i.e., quality of mother–child relationships and distant mothering). We found that mothers who recalled a better quality of pre-separation mother–child relationship tended to remotely parent their children through mobile phones in a more positive manner, which aligns with prior findings [45,46,47]. This may relate to mothers’ cognitive–affective processes. As Taraban and Shaw conclude in their theoretical model [31], a more cohesive relationship between mothers and children provides a positive feedback loop where mothers gain self-efficacy as a parent, pose a positive appraisal or expectation towards their children, and feel rewarded. Our results may align with the qualitative study by To and their colleagues [22], in which cognitions and emotions of migrant mothers motivated them to demonstrate higher levels of responsivity, proactive and directive control, autonomy granting, and lower levels of psychological and harsh punitive control from a distance. However, when migrant mothers establish a relinquishing and despondent self-cognition regarding motherhood, based on negative mother–child interactions, they may discard their childcare responsibility and decrease their engagement in mothering [81].

Moreover, this study found that mothers who reported more positive parenting also perceived a higher concurrent quality of the mother–child relation. This aligns with previous studies confirming the positive association between favorable maternal parenting and the strength of mother–child bonds [36,58,68]. A positive interpretation of this association can be framed within the context of how responsive communication practices by mothers reshape children’s perceptions of their relationships. Specifically, children of migrant mothers, despite the physical distance and absence, may perceive such communication as an indicator of care and emotional support, rather than parental rejection [82]. Furthermore, many mothers in this context may adopt more lenient parenting strategies (e.g., less frequent physical and psychological control) due to feelings of guilt over their absence [8]. This shift in parenting practices may help to reduce mother–child conflicts and foster a more harmonious relationship [83], which was reflected in the indicators of good relationship quality in our study.

### 4.2. Mediating Effects of Distant Mothering and Post-Separation Mother–Child Relationship Quality for Child Adjustment

The main aim of this study was to examine the serial mediating roles of positive distant mothering and post-separation mother–child cohesion between pre-separation cohesion and child adjustment. Partially inconsistent with our hypotheses, rather than serial mediating effects, simple mediating effects were found for child prosocial behavior and externalizing problems. Specifically, post-separation mother–child cohesion showed an individual full mediation effect between pre-separation cohesion and child prosocial behavior, while positive distant mothering demonstrated an individual full mediation effect between pre-separation cohesion and child externalizing problems. Notably, the (in)direct paths between pre-separation cohesion and each child adjustment indicator were different to some extent, which aligns with the studies that distinguish how family functioning individually explains child prosocial conduct and internalizing and externalizing difficulties [84,85]. Two reasons may explain the lack of serial mediation effects in this study. First, the small sample size likely reduced the statistical power necessary to detect such intricate relationships. Alternatively, the distinct mediation effects found for child prosocial behavior and externalizing problems suggest that pre-separation cohesion may influence different aspects of child adjustment through separate pathways, rather than through a serial mediation process.

Regarding child social behavior, our findings indicate that post-separation mother–child cohesion seemed to fully mediate the association between pre-separation mother–child cohesion and child prosocial behavior because there was no direct effect of pre-separation mother–child cohesion on prosocial behavior. This means that the association between pre-separation and prosocial behavior can be fully explained by current (post-separation) cohesion. As we expected, despite the general decline in mother–child relationship quality after separation, mothers who perceived a higher quality of pre-separation mother–child relationship showed a more cohesive relationship with their LBC, which seemingly increased the prosocial behavior of the child. The result regarding the decline in mother–child relationship quality supports the view that mothers’ migration can negatively impact the mother–child bond [41,42]. The change in mother–child relationship quality may be exacerbated by delayed family reunions due to human mobility restrictions during China’s COVID-19 pandemic [86]. When it comes to the mediation model, consistent with previous research on the strong link between earlier and later mother–child relationship quality [32,43,44], mother–child relationship quality followed a predictable and consistent trend over time (pre- versus post-separation). That is to say, mothers kept their relative position within the group of mothers regarding the mother–child relationship quality from the togetherness period to the separation period. Besides their families’ efforts, one reason for the high correlation between pre- and post-separation cohesion is the generally shorter duration of being separated for young LBC, compared to their adolescent counterparts who, on average, experience a separation of six years [19]. Another reason may be that according to mothers’ reports in the current study, most mothers chose to leave their children after the age of 3, thus after the critical period for children to develop secure attachment, according to Bowlby’s theory [66]. Secure or insecure relationships between parent and child have long-term sustainability [44]. Also, a systematic measurement error [87] may have occurred, since the quality of mother–child relationships was assessed by only one informant, i.e., the mother herself, at only one measurement point.

When it comes to the positive associations between the quality of current post-separation mother–child relationships and child prosocial behavior in the mediation model, our result supported previous behavioral evidence [54,56]. For the social factor related to the prosocial behavior mechanism, developmental psychologists emphasize that children’s experience of interpersonal relationships, especially with parents, is important to promote young children’s prosocial conduct like helping, sharing, and comforting [88]. The process may be facilitated by cultivating children’s internalized models of relationships (i.e., secure attachment) that value and prioritize reciprocal interactions and empathetic engagement with others [89]. However, since there is no direct association between pre-separation relationship quality and prosocial behavior, the explanation based on attachment theory is insufficient. An alternative explanation may be based on social learning theory, where positive mother–child interactions in video/audio calls model young children how to act prosocially [90].

Concerning child externalizing difficulties, the results showed that positive distant mothering played a full mediating role in the association between pre-separation cohesion and this child outcome, and the direct association between pre-separation cohesion and externalizing behavior remained insignificant whether or not positive distant mothering was added. In other words, pre-separation cohesion itself could not be directly linked to child externalizing difficulties, and positive distant mothering might completely channel the effect of pre-separation cohesion on externalizing problems. As we hypothesized based on previous findings [58], a higher quality of pre-separation mother–child relationship was associated with more positive distant mothering, which might go together with fewer child externalizing difficulties. We can explain this mediating effect by regarding the recalled pre-separation cohesion as a component of maternal well-being [91]. That is to say, migrant mothers might be prone to be more reflective and self-controlled in distant parenting under a satisfying and fraught mindset regarding childrearing. As for the path between the mediator and dependent variable, positive distant mothering (i.e., mothering with higher levels of positive behavioral control and lower levels of psychological and harsh punitive control) was associated with fewer externalizing problems in young children, which is consistent with the findings from the review studies by Pinquart [61] and McKee et al. [92]. McKee et al. delineated that parents’ contentious and hostile behaviors towards their children situate them in a vicious reinforcement circle, in which children are socialized to replicate parents’ hostility in non-domestic interactions, and both of them perpetuate these tendencies [92]. Children can develop self-regulation and compliance when their parents frequently use firm, proactive, and consistent behavioral control like monitoring, and these characteristics in children protect them from aggressive and oppositional propensities [92].

For child internalizing difficulties, we only found a significant total effect of the model where pre-separation mother–child cohesion was expected to link internalizing problems through post-separation cohesion. This result might be explained by the strong association between pre- and post-separation relationship quality, while each path between pre-/post-separation cohesion and internalizing symptoms was insignificant. Theoretically, mother–child relationship quality is interpreted as one of the possible determinants of child internalizing difficulties. High relationship quality may support children to develop a positive self-concept, higher self-esteem, better emotional regulation skills, and effective coping strategies, which are a salient buffer against psycho-emotional difficulties like anxiety and depression [93]. However, our study did not show a significant association between mother–child cohesion and child internalizing symptoms. Three alternative explanations can account for this result. First, the mechanisms connecting mother–child relationship quality and child internalizing problems depend more on children’s own perceptions and interpretations [94]. We used mother-reported mother–child cohesion, but children often have a different perspective about their mothers’ [95]. Child-perceived relationship quality with their mothers would be a more optimal predictor. For instance, the Four Field Map Task can be an option, with which children place their family members including their mother on a circle map with four rings to indicate the closeness of the relationships between the figures and them based on each figure’s distance to the center (representing the child themselves) [96]. Second, given the young age of the children in this study, the effect of mother–child relationship quality on child internalizing symptoms may take a longer time to emerge. Because in early childhood, children are in the cognitive pre-mature phase to acquire greater proficiency in recalling and ruminating about negative and miserable events (e.g., mother–child conflicts and family separation) for later habituated cognitive patterns [97,98], the accumulative process cannot be detected with a cross-sectional research design. Third, in the aspect of interpersonal relationships’ influence on internalizing, grandparent–child relationship quality in these work-separated families may contribute more to child internalizing symptoms over mother–child relationship quality. A higher quality of grandparent–grandchild relationship is conducive to reducing the risks of loneliness and depression among LBC [99,100]. In the context of parental absence, grandparents usually become the primary caregivers who provide emotional support to LBC. Even before parental migration or in non-LBC families, grandparenting is quite common in Chinese families [101]. Therefore, the process of grandparent–child bonds may be especially profound for children’s emotional adaption, but we here focused on the mother–child relationship only.

### 4.3. Strengths and Limitations

The current findings underscore the importance of a continuous high-quality mother–child bond and favorable mothering through ICTs for work-separated families in China. Our study further extends the process model of parenting by bringing evidence regarding this situational parenting and migration backgrounds to manifest the compatibility of the general ecological theory [30]. Building on previous qualitative research on relationship maintenance of separated families [8,10], our findings provide larger-scale evidence to the exploration regarding long-distance parenting towards young children and family well-being. In addition, the multi-informant approach used in our study displays a broader picture of LBC’s adjustment behaviors in both domestic and preschool situations.

We acknowledge that our study has several limitations and there are some potential extensions of the current work. First, as we explained, some effects in our analysis model required greater statistical power. Additionally, due to the sample size restriction, we were unable to include several important covariates (e.g., grandparenting) in the model to conserve statistical power. Further research on the pathways between pre- and post-separation mother–child relationships, distant mothering, and child adjustment—using a larger sample size and incorporating more key covariates—is recommended. Second, because of the cross-sectional data and social desirability bias in questionnaire answering, the validity of pre-separation mother–child relationship quality and associations between the variables could be questioned. For instance, the recalled relationship quality might differ from the actual pre-separation relationship quality as mothers who experienced earlier separations might have a less accurate recollection of the mother–child bond before the separation. The deviation might also stem from maternal mental health, which might influence the cognitive process of how they reminisce or perceive their interactions with the offspring [102,103]. Especially during the COVID-19 pandemic, migrant workers in China faced increasing risks of psychological issues due to sudden unemployment, loss of financial sources, and enforced quarantine [104]. Migrant mothers of preschool-age children were struggling more with the trade-off of parenting and employment compared to fathers in this hard time [52]. Longitudinal research and additional mothers’ reports on mental health will enable future studies to avoid confounding effects and unravel the fine-grained associations or causal links between family functioning and child development. Third, our data and analyses did not allow us to specify the associations between each distant mothering dimension and each child adjustment outcome. For example, a high level of maternal directive control was regarded as one component of positive distant mothering with an acceptable but low loading. This kind of parental behavioral control may have a curvilinear association with child externalizing symptoms [105]. Differentiating between parenting dimensions in future studies may offer more tailored implications for supporting children with specific difficulties. Fourth, given the limited number and specific types of households in this study, caution is needed when generalizing the findings to other families of left-behind children with one-parent migration, as well as to families from different cultures and locations.

## 5. Conclusions and Implications

In this study, we found that post-separation mother–child cohesion played a mediating role in the association between pre-separation mother–child cohesion and child prosocial behavior. By contrast, the association between pre-separation mother–child cohesion and child externalizing problems was mediated by positive distant mothering. The qualities of pre- and post-separation mother–child relationships and positive distant mothering were positively related to each other, which formed as a constituent of beneficial family dynamics.

The evident mediating role of current mother–child cohesion for child prosocial behavior indicates that even if mothers and children have difficulty in developing close relationships before separation, it is still possible and worthwhile to promote children’s altruistic tendencies by supporting migrant mothers to improve a cohesive relationship later. Accordingly, practitioners working with families of LBC can help migrant mothers figure out the potential threats to their mother–child bonds. For instance, based on our detected mechanism between distant mothering and mother–child cohesion, practitioners can provide alternatives for practices that may put pressure on a positive parent–child relationship, such as psychological control or physical punishment from a distance. Further, if mothers’ mental health appears to be undermining a positive mother–child bond, such as with depression, then social workers can help these mothers access local non-profit psychological counseling services for migrant populations.

In addition, it is interesting to observe the mediating effect of positive distant mothering for child externalizing problems, indicating that positive distant mothering can function as a focus of prevention and intervention programs for decreasing child problem behaviors. The questionnaire that was used in this study to assess mobile phone parenting, the MPPPQ, presents a scheme with six types of distant mothering practices that migrant parents can consult when they want to interact with their children through mobile phones or tablets. Increasingly empirical evidence has confirmed the effectiveness of parent education programs specifically targeting socially disadvantaged mothers, demonstrating their substantial potential for promoting positive parenting practices [106,107]. For migrant workers under the burden of time and workload, online interventions or the approach through mobile phone applications can be a better option.

## Figures and Tables

**Table 1:** Demographic Information and Descriptive Statistics.

Variables	*N*	Percentage
**Child s** **ex**		
Girl	83	44.86
Boy	102	55.14
**Family annual income (CNY)**		
35,000 and below	10	5.41
35,001–55,000	21	11.35
55,001–75,000	43	23.24
75,001–95,000	63	34.05
95,001–115,000	31	16.76
115,001 and more	17	9.19
**Father–child telecommunication frequency**		
Less than once a week	54	29.19
Once a week	40	21.62
Twice a week	39	21.08
Three times a week	21	11.35
Four times a week	15	8.11
Five times a week	14	7.57
Six times a week and more	2	1.08
**Mother–child separation duration**		
Fewer than once year	65	35.14
One year	58	31.35
Two years	40	21.62
Three years	19	10.27
Four years and more	3	1.62
	**Mean**	***SD***
Child age	4.23	1.01
Mother age	31.94	3.76
Mother’s completed educational years	9.75	1.87
Teacher-reported prosocial behavior	6.92	2.06
Teacher-reported internalizing problems	5.42	2.99
Teacher-reported externalizing problems	6.93	2.89
Grandparent-reported prosocial behavior	7.10	2.06
Grandparent-reported internalizing problems	5.01	2.63
Grandparent-reported externalizing problems	5.40	2.95
Composed prosocial behavior	7.01	1.69
Composed internalizing problems	5.21	2.43
Composed externalizing problems	6.16	2.31
Recalled pre-separation mother–child cohesion	61.97	7.39
Current post-separation mother–child cohesion	59.40	7.62
Maternal responsivity	3.85	0.74
Maternal proactive control	3.70	0.95
Maternal directive control	3.37	0.90
Maternal autonomy granting	3.65	0.92
Maternal psychological control	2.31	0.93
Maternal harsh punitive control	1.63	0.53

*Note. SD* = standard deviation; 1 CNY  ≈  0.14 EUR.

**Table 2:** Correlations Among the Key Variables.

	1	2	3	4	5	6	7	8	9	10	11	12	13	14	15	16
1. Child age																
2. Child sex	−0.15 *															
3. Mother’s completed educational years	0.03	−0.05														
4. Family income	0.10	−0.05	0.22 **													
5. Father–child telecommunication frequency	−0.06	−0.09	0.01	0.07												
6. Duration of mother–child separation	0.55 ***	−0.04	−0.05	0.03	−0.07											
7. Composed prosocial behavior	0.03	−0.08	0.14	0.19 **	0.16 *	0.00										
8. Composed internalizing problems	0.03	−0.16 *	−0.17 *	−0.18 *	−0.15 *	−0.02	−0.27 ***									
9. Composed externalizing problems	0.00	0.02	−0.13	−0.11	−0.13	0.01	−0.22 **	0.47 ***								
10. Recalled pre-separation mother–child cohesion	0.08	−0.02	0.04	0.14	0.18 *	0.05	0.21 **	−0.32 ***	−0.28 ***							
11. Current post-separation mother–child cohesion	0.05	−0.09	0.03	0.17 *	0.18 *	0.04	0.28 ***	−0.32 ***	−0.26 ***	0.75 ***						
12. Maternal responsivity	0.16 *	−0.04	0.22 **	0.13	0.04	0.04	0.23 **	−0.17 *	−0.24 **	0.30 ***	0.29 ***					
13. Maternal proactive control	0.03	0.00	0.15 *	0.05	0.00	0.07	0.11	−0.16 *	−0.20 **	0.24 **	0.25 **	0.36 ***				
14. Maternal directive control	0.09	−0.04	0.13	0.13	0.00	0.03	0.06	−0.11	−0.14	0.07	0.08	0.26 **	0.35 ***			
15. Maternal autonomy granting	0.02	0.08	0.23 **	0.20 **	0.01	0.12	0.16 *	−0.23 **	−0.18 *	0.19 *	0.20 **	0.50 ***	0.35 ***	0.35 ***		
16. Maternal psychological control	−0.22 **	−0.04	−0.11	−0.10	−0.08	−0.22 **	−0.09	0.17 *	0.12	−0.10	−0.17 *	−0.44 ***	−0.36 ***	−0.24 **	−0.35 ***	
17. Maternal harsh punitive control	−0.06	−0.02	−0.18 *	−0.27 ***	−0.03	−0.05	−0.07	0.17 *	0.19 **	−0.25	−0.28 ***	−0.49 ***	−0.38 ***	−0.28 ***	−0.46 ***	0.41 ***

*Note. p*-values: * *p* < 0.05, ** *p* < 0.01, *** *p* < 0.001. Child sex: 0 = *girl*, 1 = *boy*. Spearman correlation was used for associations involving child sex while the other bivariate associations among the variables were tested by Pearson correlation.

**Table 3:** Unstandardized and Standardized Coefficients from the Structural Model Regarding the (In)direct Paths of Positive Distant Mothering and Post-Separation Mother–Child Cohesion on the Associations Between Pre-Separation Mother–Child Cohesion and Child Outcomes.

	*B*	*SE*	*β*	*p*	95% CI Lower	95% CI Upper
**Mediators**						
pre-separation cohesion–mothering	0.02	0.01	0.29	<0.000 ***	0.15	0.44
mothering–post-separation cohesion	10.96	0.91	0.14	0.031 *	0.02	0.26
pre-separation cohesion–post-separation cohesion	0.72	0.05	0.70	<0.000 ***	0.63	0.77
**Prosocial behavior**						
mothering–prosocial behavior	0.24	0.30	0.08	0.418	−0.11	0.26
post-separation cohesion–prosocial behavior	0.05	0.02	0.24	0.031 *	0.03	0.45
pre-separation cohesion–prosocial behavior	−0.01	0.02	−0.03	0.783	−0.24	0.18
indirect path 1	0.00	0.00	0.01	0.150	0.00	0.02
total effect 1	0.24	0.29	0.06	0.424	−0.20	0.31
indirect path 2	0.01	0.01	0.02	0.427	−0.03	0.08
total effect 2	0.00	0.02	−0.01	0.945	−0.22	0.20
indirect path 3	0.04	0.02	0.16	0.033 *	0.02	0.31
total effect 3	0.03	0.02	0.14	0.077	−0.01	0.28
**Internalizing problems**						
mothering–internalizing problems	−0.52	0.41	−0.11	0.205	−0.28	0.06
post-separation cohesion–internalizing problems	−0.06	0.03	−0.17	0.094	−0.38	0.03
pre-separation cohesion–internalizing problems	−0.04	0.03	−0.12	0.241	−0.32	0.08
indirect path 4	0.00	0.00	−0.01	0.203	−0.02	0.00
total effect 4	−0.56	0.41	−0.24	0.169	−0.48	0.01
indirect path 5	−0.01	0.01	−0.03	0.227	−0.09	0.02
total effect 5	−0.05	0.03	−0.15	0.139	−0.35	0.05
indirect path 6	−0.04	0.02	−0.12	0.097	−0.26	0.02
total effect 6	−0.08	0.02	−0.24	0.001 **	−0.38	−0.10
**Externalizing problems**						
mothering–externalizing problems	−0.95	0.41	−0.22	0.021 *	−0.39	−0.04
post-separation cohesion–externalizing problems	−0.01	0.03	−0.04	0.709	−0.25	0.17
pre-separation cohesion–externalizing problems	−0.05	0.03	−0.16	0.130	−0.37	0.05
indirect path 7	0.00	0.00	0.00	0.712	−0.01	0.01
total effect 7	−10.00	0.41	−0.38	0.015 *	−0.63	−0.13
indirect path 8	−0.02	0.01	−0.06	0.046 *	−0.12	−0.00
total effect 8	−0.07	0.03	−0.22	0.038 *	−0.43	−0.02
indirect path 9	−0.01	0.02	−0.03	0.709	−0.18	0.12
total effect 9	−0.06	0.02	−0.19	0.012 *	−0.33	−0.04

*Note.* Indirect path 1: pre-separation cohesion–mothering–post-separation cohesion–prosocial behavior; indirect path 2: pre-separation cohesion–mothering–prosocial behavior; indirect path 3: pre-separation cohesion–post-separation cohesion–prosocial behavior; indirect path 4: pre-separation cohesion–mothering–post-separation cohesion–internalizing problems; indirect path 5: pre-separation cohesion–mothering–internalizing problems; indirect path 6: pre-separation cohesion–post-separation cohesion–internalizing problems; indirect path 7: pre-separation cohesion–mothering–post-separation cohesion–externalizing problems; indirect path 8: pre-separation cohesion–mothering–externalizing problems; indirect path 9: pre-separation cohesion–post-separation cohesion–externalizing problems. The numbers for the total effects correspond to the numbers for the indirect paths. *SE* = standard error; CI = confidence interval. *p*-values: * *p* < 0.05, ** *p* < 0.01, *** *p* < 0.001.

## Data Availability

The data that support the findings of this study are available on request from the corresponding author, Ruwen Liang. The data are not publicly available due to their containing information that could compromise the privacy of research participants.
